# Deep learning with language models improves named entity recognition for PharmaCoNER

**DOI:** 10.1186/s12859-021-04260-y

**Published:** 2021-12-17

**Authors:** Cong Sun, Zhihao Yang, Lei Wang, Yin Zhang, Hongfei Lin, Jian Wang

**Affiliations:** 1 School of Computer Science and Technology, Dalian University of Technology, Dalian, China; 2 Beijing Institute of Health Administration and Medical Information, Beijing, China

**Keywords:** Named entity recognition, NER, Language model, BERT, Text mining

## Abstract

**Background:**

The recognition of pharmacological substances, compounds and proteins is essential for biomedical relation extraction, knowledge graph construction, drug discovery, as well as medical question answering. Although considerable efforts have been made to recognize biomedical entities in English texts, to date, only few limited attempts were made to recognize them from biomedical texts in other languages. PharmaCoNER is a named entity recognition challenge to recognize pharmacological entities from Spanish texts. Because there are currently abundant resources in the field of natural language processing, how to leverage these resources to the PharmaCoNER challenge is a meaningful study.

**Methods:**

Inspired by the success of deep learning with language models, we compare and explore various representative BERT models to promote the development of the PharmaCoNER task.

**Results:**

The experimental results show that deep learning with language models can effectively improve model performance on the PharmaCoNER dataset. Our method achieves state-of-the-art performance on the PharmaCoNER dataset, with a max F1-score of 92.01%.

**Conclusion:**

For the BERT models on the PharmaCoNER dataset, biomedical domain knowledge has a greater impact on model performance than the native language (i.e., Spanish). The BERT models can obtain competitive performance by using WordPiece to alleviate the out of vocabulary limitation. The performance on the BERT model can be further improved by constructing a specific vocabulary based on domain knowledge. Moreover, the character case also has a certain impact on model performance.

## Background

Effectively recognizing biomedical entities from texts is of great value to biomedical research [1]. With the rapid increase in literature scale, it is no longer possible to recognize biomedical entities from texts through manual annotations. Therefore, using natural language processing (NLP) methods to recognize these entities automatically has attracted plenties of attention. Biomedical named entity recognition (BioNER) is such an NLP task. The importance of biomedical entity recognition motivated several shared tasks, such as the CHEMDNER track [2], the SemEval challenge [3], and the i2b2 challenge [4]. Most biomedical and clinical NLP studies are conducted on English texts, while only few works are done using non-English texts. However, it is essential to note that many texts are published in non-English, especially in clinical case reports, mostly written in the native language. Therefore, it is necessary to recognize biomedical named entities in non-English literature. PharmaCoNER [5] is the first BioNER challenge devoted to recognizing chemical and protein entities from biomedical literature in Spanish. The primary purpose is to promote non-English BioNER tools, determine the best performing method, and compare the systems that obtain state-of-the-art (SOTA) performance [5]. The PharmaCoNER challenge consists of two sub-tracks: NER offset and entity classification and concept indexing. In this work, we only focus on the first sub-track.

In the previous works, the implementation of BioNER methods [6, 7] mainly depended on feature engineering, i.e., using various NLP tools and external resources to construct features. This is a skill-dependent and laborious task. To overcome the limitations, neural network methods with automatic feature learning abilities have been widely proposed [8–11]. These methods use pre-trained word embeddings [12–14] to learn the semantic information of each word and combine neural network models such as LSTMs and CNNs to encode the context information to implement BioNER tasks. However, once the word embeddings are pre-trained, the word will be mapped to a specific vector, and therefore, the word embeddings can only learn context-independent representations. Recently, neural language models [15–17] have improved the performance of NLP methods to a new level. Unlike traditional word embeddings such as Word2Vec [12, 13] and GloVe [14], the word embeddings pre-trained by language models depend on the context. Therefore, the same word can have different semantic information in different contexts. Due to the great success of language models, it has gradually developed into the mainstream method to implement BioNER tasks.

During the PharmaCoNER challenge, a total of 22 teams participated in the NER sharing task, and the top three models ranked by performance were all based on language models. Specifically, Xiong et al. [18] achieved the best performance, reaching an F1-score of 91.05%. In their approach, they first employed Multilingual BERT [17] as language representations, and then combined the character-level representation, part-of-speech (POS) representation and word shape representation of each word to the BERT representation. Finally, a conditional random field (CRF) layer is appended to these representations for the BioNER task. Stoeckel et al. [19] obtained the second-best performance. They trained a BiLSTM-CRF sequence tagger with stacked pooled contextualized embeddings, word embeddings and sub-word embeddings using the FLAIR framework [16, 20]. Sun et al. [21] leveraged Multilingual BERT [17] and BioBERT [22] to implement solutions for the PharmaCoNER challenge, and their solutions obtained third-place performance. From the PharmaCoNER challenge, neural language models, especially BERT, obtain SOTA performance in the NER task. Compared with other methods (i.e., CRF and BiLSTM-CRF), neural language models can effectively learn latent context information and improve model performance. BERT has become the most representative language model with its powerful performance and abundant resources among these language models. Leveraging existing BERTs to obtain SOTA performance has important research implications for non-English NER tasks with fewer resources. Although some BERT models have been employed during the PharmaCoNER challenge, there are still many representative BERT models in the NLP community that have not been explored. In this article, we compare and explore the impact of these BERTs on the PharmaCoNER corpus.

## Methods

### PharmaCoNER

The goal of the PharmaCoNER task is to recognize chemical and protein entities from a given input sentence or article in Spanish. The PharmaCoNER corpus is a partial collection of the Spanish Clinical Case Corpus (SPACCC). It contains 1000 clinical cases, of which 500 are used as the training set, 250 as the development set, and 250 as the test set. Each clinical case is composed of two standoff-style annotation documents, i.e., a ‘txt’ document used for describing the clinical record, and an ‘ann’ document used for tagging biomedical entities of the case. In this work, the input of the BERT model is sentences, which are obtained by splitting the documents from the PharmaCoNER corpus according to sentence symbols (e.g. ‘.!?’). There are three types of entities to be evaluated in the PharmaCoNER corpus, namely ‘NORMALIZABLES’ entities, ‘NO_NORMALIZABLES’ entities, and ‘PROTEINAS’ entities. The ‘NORMALIZABLES’ entities represent chemical entities that can be manually standardized as unique concept identifiers (primarily SNOMED-CT). The ‘NO_NORMALIZABLES’ entities represent chemical entities that cannot be manually standardized as unique concept identifiers. The ‘PROTEINAS’ entities denote protein and gene entities that can be annotated according to the BioCreative GPRO track guidelines [23], and it also includes peptides, peptide hormones and antibodies. Furthermore, the PharmaCoNER corpus also contains a type of ‘UNCLEAR’ entities, which denote general substance entities of clinical or biomedical relevance, including pharmaceutical formulations, general treatments, chemotherapy programs, vaccines. The ‘UNCLEAR’ entities are not used to evaluate the PharmaCoNER task but as additional annotations of biomedical relevance. Table 1 illustrates the statistical information of the PharmaCoNER corpus.

**Table 1:** The statistical information of the PharmaCoNER corpus

Set	Training	Development	Test	Total
Documents	500	250	250	1000
Sentences	7003	3454	3403	13860
NORMALIZABLES	2304	1121	973	4398
NO_NORMALIZABLES	24	16	10	50
PROTEINAS	1405	745	859	3009
UNCLEAR	89	44	34	167

**Fig. 1:**
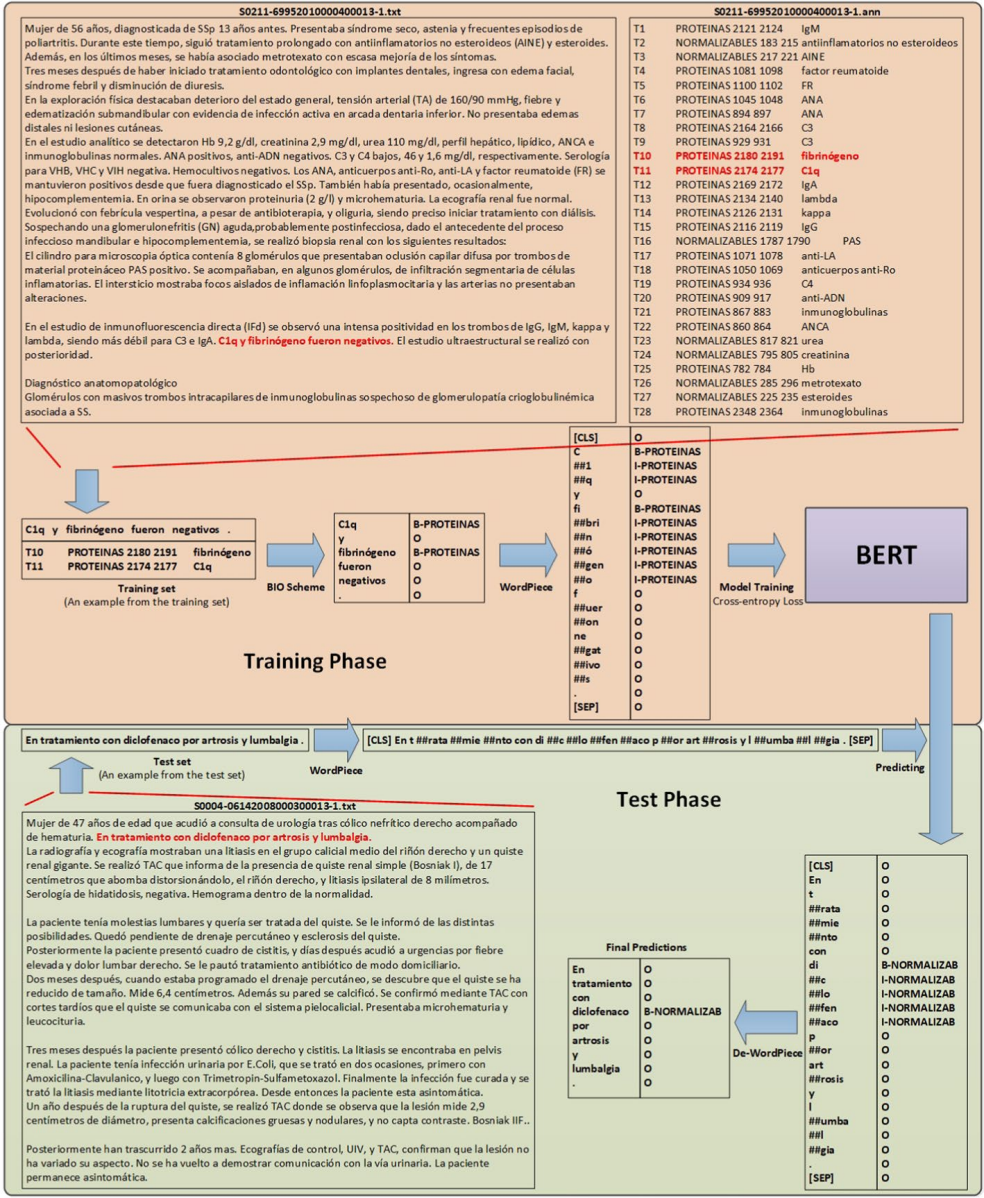
The processing flowchart of our approach

Figure 1 shows the flowchart of our approach. We use Begin, Inside, Outside (BIO) scheme to tag the input sequence and formulate the PharmaCoNER task as a multi-class classification problem. Take the “C1q y fibrinógeno fueron negativos.” sentence from the training set as an example. Because ‘C1q’ and ‘fibrinógeno’ are ‘PROTEINAS’ entities and other tokens are not biomedical entities, the corresponding BIO tags can be expressed as “B-PROTEINAS O B-PROTEINAS O O O”. Moreover, BERT uses WordPiece to alleviate the out-of-vocabulary (OOV) problem. Therefore, in the training phase, the input sentence needs to be further processed by the WordPiece tokenizer, and the final processed tokens are used as the model input. Correspondingly, the BIO tags predicted by the BERT model also need to be processed by De-WordPiece to obtain the BIO tags of the original sentence in the test phase. Formally, given an input sequence *S* = $\{w_1,\cdots ,w_i,\cdots ,w_n\}$, the objective of PharmaCoNER is to estimate the probability $P(t|w_i)$, where $w_i$ is the *i*-th word/token, *T* = {O, B-NORMALIZABLES, I-NORMALIZABLES, B-NO_NORMALIZABLES, I-NO_NORMALIZABLES, B-PROTEINAS, I-PROTEINAS}, $t \in T$, and $1 \le i \le n$.

The performance on the PharmaCoNER challenge is measured with the precision (P), recall (R), and micro-averaged F1-score (F1). The formulas are:

1 $$\begin{aligned}&P = \frac{TP}{TP+FP} \end{aligned}$$

2 $$\begin{aligned}&R = \frac{TP}{TP+FN}\end{aligned}$$

3 $$\begin{aligned}&F1 = \frac{2\cdot P \cdot R}{P+R} \end{aligned}$$

where *TP*, *FP* and *FN* denote true positive, false positive, and false negative, respectively.

**Fig. 2:**
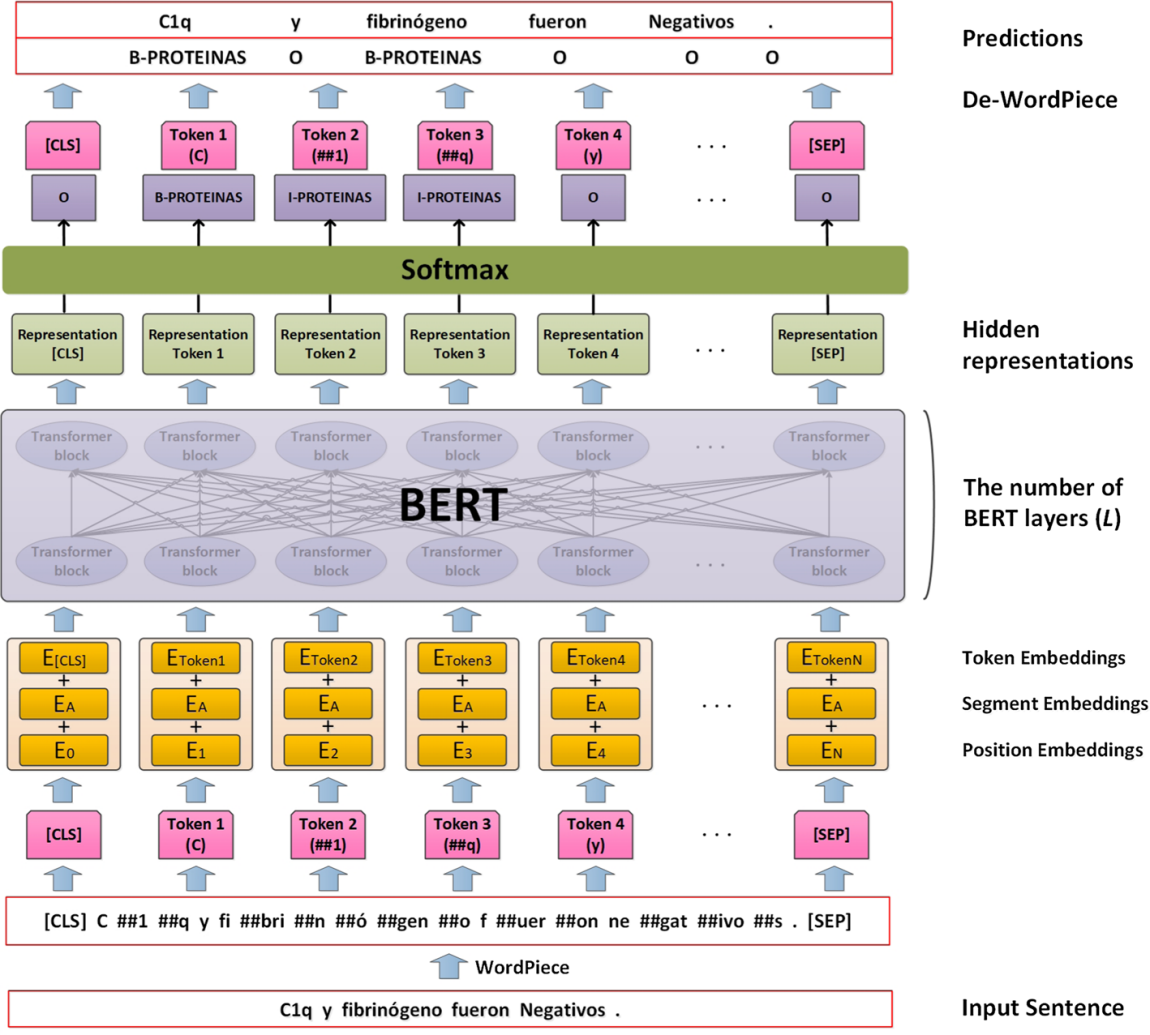
The architecture of the BERT model

### BERT architecture

BERT [17], which stands for bidirectional encoder representations from Transformers [24], is a contextualized word representation model. It aims to pre-train a deep bidirectional context representation based on the left and right contexts of all layers. Because BERT has been widely used in various NLP tasks, and our implementation is effectively identical to the original, we refer readers to read the original paper [17] for more details about BERT. In this work, we only use the BERT model to implement solutions for the PharmaCoNER task. Figure 2 shows the architecture of the BERT model on the PharmaCoNER task. The BERT model first uses the WordPiece tokenizer [25] to tokenize the input sentence and adds unique tokens ‘[CLS]’ and ‘[SEP]’ to indicate the head and tail of the sentence. Then, the representation of each token in the input sentence is constructed by summing the corresponding token, segment, and position embeddings, and further fed into multiple layers of Transformers. Note that the segment embeddings can use different values to distinguish whether the input sequence is a single sentence or a sentence pair. We only use the single sentence as the model input in the experiments, so the segment embeddings share the same value. Afterward, the hidden representations of the *L*-th layer of the BERT model (the number of BERT layers is denoted as *L*) are used by the softmax function to predict token classifications. Finally, the BERT model predicts the BIO tags of the original sentence after the De-WordPiece process.

According to different scales, BERT provides two model sizes: BERT$_{BASE}$ and BERT$_{LARGE}$. For each model size, the number of layers *L*, the hidden size *H*, and the number of self-attention heads *A* are listed as follows:

- BERT$_{BASE}$: *L*=12, *H*=768, *A*=12, Total Parameters=110M.
- BERT$_{LARGE}$: *L*=24, *H*=1024, *A*=16, Total Parameters=340M.

Due to the limitation of computing resources, the BERT$_{BASE}$ model is more widely used than the BERT$_{LARGE}$ model. Therefore, we mainly explore the BERT$_{BASE}$ model in this research.

### Pre-training procedure

**Fig. 3:**
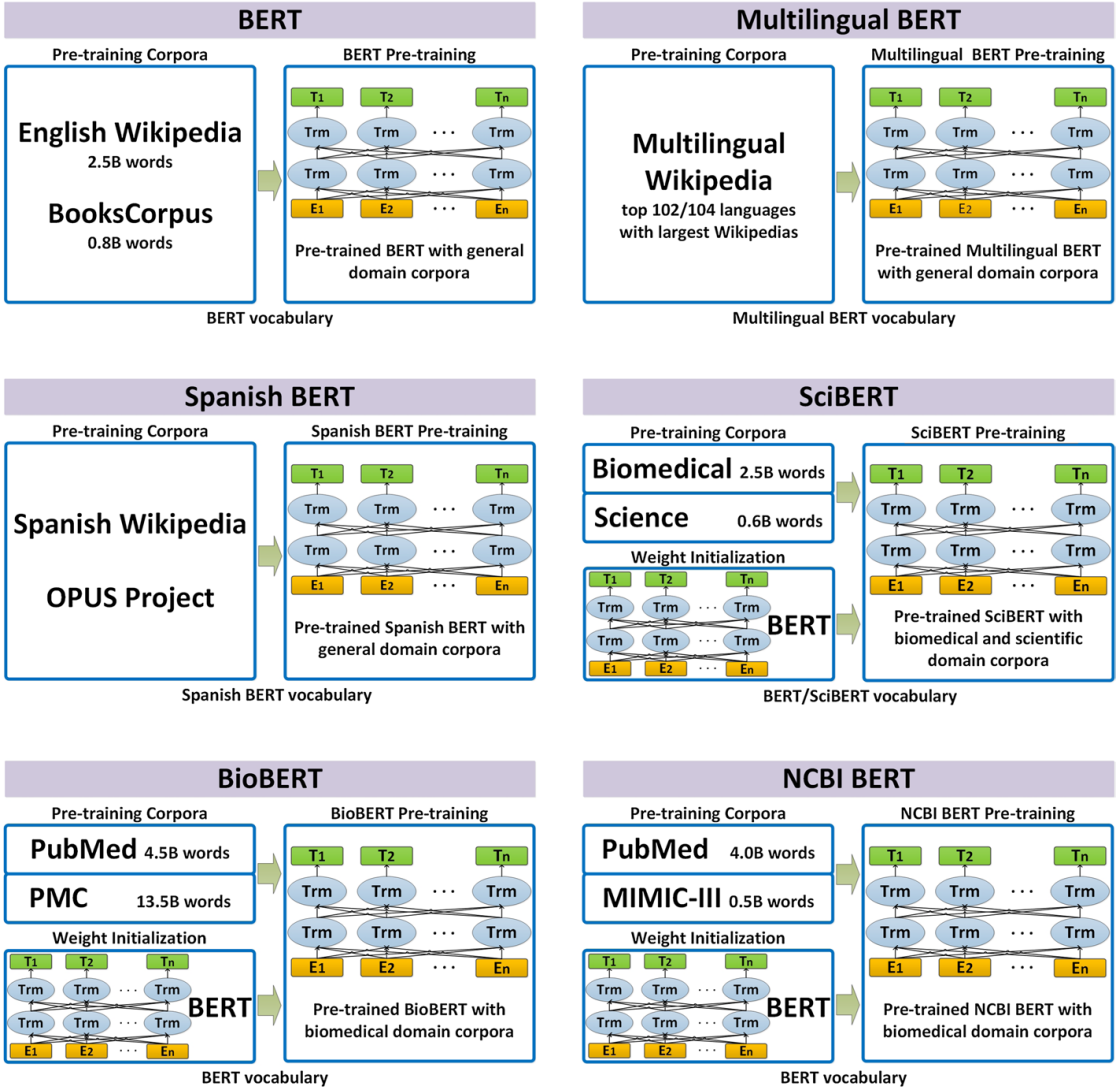
Overview of the pre-training process of various BERT models. Panels adapted from Lee et al. [22]

BERT is pre-trained using two unsupervised prediction tasks, masked language model [26] and next sentence prediction. The masked language model predicts randomly masked words in the input sequence and, therefore, can be used to learn bidirectional representations. The next sentence prediction can be employed to learn the relationship between sentences. As a general-purpose language representation model, the original BERT model was pre-trained on English Wikipedia (2.5B words) and BooksCorpus (0.8B words) [27]. However, biomedical texts contain a large number of biomedical entities (e.g., ‘3-(4,5-dimethylthiazol-2yl)-2,5-diphenyltetrazolium bromide’, ‘nitrato de plata’), which are generally only understood by specific researchers. Therefore, the performance on models designed for general English understanding may not be satisfactory. To solve this problem, biomedical researchers use the corpus of their domain to pre-train the BERT model. As a result, many different BERT models have appeared in the NLP community based on diverse pre-training corpus or methods. Figure 3 illustrates the representative BERT models, and Table 2 shows the detailed comparison of these models.

**Table 2:** Comparison of existing BERTs

Model	Corpus combination	Vocabulary
BERT(Cased)	Wiki+Books(Original)	BERT
BERT(Uncased)	Wiki+Books(Original)	BERT
NCBI BERT(+P,Uncased)	Original+PubMed	BERT
NCBI BERT(+P+M,Uncased)	Original+PubMed+MIMIC-III	BERT
Spanish BERT(Cased)	Original+Spanish Wikipedia+OPUS	Spanish BERT
Spanish BERT(Uncased)	Original+Spanish Wikipedia+OPUS	Spanish BERT
MultiBERT(Cased)	Multilingual Wikipedia	MultiBERT
MultiBERT(Uncased)	Multilingual Wikipedia	MultiBERT
SciBERT(BertVoc,Cased)	Original+Biomedical+Scientific	BERT
SciBERT(BertVoc,Uncased)	Original+Biomedical+Scientific	BERT
SciBERT(SciVob,Cased)	Original+Biomedical+Scientific	SciBERT
SciBERT(SciVob,Uncased)	Original+Biomedical+Scientific	SciBERT
BioBERTv1.0(+P,Cased)	Original+PubMed	BERT
BioBERTv1.0(+PMC,Cased)	Original+PMC	BERT
BioBERTv1.0(+P+PMC,Cased)	Original+PubMed+PMC	BERT
BioBERTv1.1(+P,Cased)	Original+PubMed	BERT

#### NCBI BERT

NCBI BERT [28] is an uncased BERT model pre-trained using biomedical domain corpora (PubMed or MIMIC-III). It uses the original BERT model to initialize the weights and further exploits its vocabulary, sequence length, and other configurations to pre-train the model. There are two versions of NCBI BERT based on BERT$_{BASE}$, namely NCBI BERT(P,Uncased) and NCBI BERT(P+M,Uncased), where ‘P’ denotes PubMed and ‘M’ denotes MIMIC-III, respectively. The NCBI BERT(P,Uncased) model was pre-trained with 5M steps on PubMed, and the NCBI BERT(P+M,Uncased) model was pre-trained with 5M steps on PubMed and 0.2M steps on MIMIC-III.

#### Spanish BERT

Spanish BERT (also called es-BERT) [29] is a BERT model pre-trained on a large Spanish general domain corpus. This BERT model is slightly different from BERT$_{BASE}$, and it has 12 transformer layers with 16 self-attention heads each layer, using 1024 as the hidden size. For pre-training Spanish BERT, the authors leveraged all the data from Spanish Wikipedia and all the sources of the OPUS Project [30] that have text in Spanish.

#### Multilingual BERT

Multilingual BERT [17] is a BERT$_{BASE}$ model pre-trained using the top 104 languages in Wikipedia, and its model structure is the same as BERT$_{BASE}$. Furthermore, Multilingual BERT uses a 110k shared WordPiece vocabulary as its vocabulary. Because the size of Wikipedia for a given language varies greatly, low-resource languages may be “under-represented” in terms of the neural network model under the assumption that languages compete for limited model capacity. To overcome this limitation, Multilingual BERT performed exponentially smoothed weighting of the data during the pre-training phase to balance the sampling of high-resource languages and low-resource languages. As a result, high-resource languages like English will be under-sampled, and low-resource languages like Icelandic will be over-sampled.

#### SciBERT

SciBERT [31] is a pre-trained contextualized language model based on BERT$_{BASE}$ to address the lack of high-quality, large-scale labeled scientific data. It exploits unsupervised pre-training on a large computer science domain and biomedical domain corpora to improve performance on downstream NLP tasks. The authors of SciBERT used the original BERT model to train SciBERT with the same configuration and size as BERT$_{BASE}$. They trained four different versions in total based on cased/uncased character and BERT/SciBERT vocabulary. The models using SciBERT vocabulary are pre-trained from scratch, while the models using BERT vocabulary are initialized from BERT weights.

#### BioBERT

BioBERT [22] is another BERT model trained on biomedical domain corpora (e.g., PubMed and PMC), and its structure is basically the same as BERT$_{BASE}$. BioBERT uses the original vocabulary of BERT$_{BASE}$ as its vocabulary, and it is a cased model. There are four versions of BioBERT based on different corpora for pre-training, namely BioBERTv1.0(+P,Cased), BioBERTv1.0(+PMC,Cased), BioBERTv1.0(+P+PMC,Cased), and BioBERTv1.1(+P,Cased), where ‘P’ means PubMed, and ‘+’ denotes a new corpus in addition to BooksCorpus and English Wikipedia. Specifically, BioBERTv1.0(+P+PMC,Cased) is a version pre-trained on 470K steps. When using both the PubMed and PMC corpora, the authors of BioBERT found that 200K and 270K pre-training steps were optimal for PubMed and PMC, respectively. Therefore, the ablated versions which were pre-trained on only PubMed for 200K steps (i.e., BioBERTv1.0(+P,Cased)) and PMC for 270K steps (BioBERTv1.0(+PMC,Cased)) were provided. Moreover, the authors also provided a BioBERT version pre-trained on PubMed for 1M steps, namely BioBERTv1.1(+P,Cased).

### Fine-tuning procedure

With minimal architectural modification, various existing BERT models can be used for downstream NLP tasks. As shown in Fig. 2, BERT in the figure represents a BERT model pre-trained using specific corpora (e.g., BioBERT, SciBERT). In this work, we use the PharmaCoNER dataset to fine-tune the BERT model. Specifically, the sentence processed by the WordPiece tokenizer is used as the input to the BERT model in the training phase. The BERT model learns the input feature of each token and dynamically tune model parameters, and then classify each token through the Softmax function. The BIO tag of each input token/word can be obtained after the De-WordPiece process. The cross-entropy loss function calculates the loss value between the predicted token tags and the ground-truth tags at the training time. Finally, as shown in Fig. 1, the fine-tuned BERT predicts the final token BIO tags based on the input test sentences in the test phase.

## Results and discussion

### Experimental settings

In the experiments, all BERTs are implemented using the transformer framework (https://github.com/huggingface/transformers) based on the PyTorch library (https://pytorch.org). For fair comparisons, we repeat the same experiment five times with the same hyper-parameters, and report the max and average precision, recall, F1-score, as well as the standard deviation. Like most participating teams, we also combined the original training set and development set as the training set. Then we randomly sampled 10% of the training set as the validation set to tune the hyper-parameters. Specifically, the training set and validation set consisted of 9411 and 1046 sentences as the input in our experiments, respectively. The test set is only used to test the model, with 3403 sentences as the model input. The detailed experimental settings are listed in Table 3. Note that the sequence length is expressed as the maximum word/token length of each sentence allowed by the model.

**Table 3:** Detailed experimental settings

Parameters	Tune range	Optimal
Sequence length	[128, 256, 300]	300
Train batch size	[8, 16, 32]	16
Dev batch size	16	16
Test batch size	16	16
Learning rate	[1e−05, 2e−05, 3e−05]	2e−05
Epoch number	[10, 20, 30, 50]	20
Warmup	0.1	0.1
Dropout	0.1	0.1

**Table 4:** Performance comparison on the PharmaCoNER dataset

Method	P (%)	R (%)	F1 (%)
Baseline-Glove [32]	83.26	81.00	82.11
Baseline-Med [32]	87.02	83.71	85.34
Sun et al. [21]	90.46	88.06	89.24
Stoeckel et al. [19]	90.79	90.30	90.52
Xiong et al. [18]	91.23	90.88	91.05
Our method (BioBERTv1.1(+P,Cased))	**92.44**	**91.59**	**92.01**

‘P’ denotes PubMed

The highest values are shown in bold

### Experimental results

Table 4 shows the experimental results in detail. The first two methods are provided by the PharmaCoNER organizers. These two methods are based on the PharmaCoNER tagger [32], a neural network (LSTM-CRF) based tool for automatically recognizing chemical and protein entities in Spanish medical texts. The Baseline-Glove used word embeddings trained by GloVe [14] on the Spanish Billion Word Corpus, and the Baseline-Med leveraged word embeddings from the Medical Word Embeddings for Spanish [33]. Baseline-Glove and Baseline-Med obtain F1-scores of 82.11% and 85.34%, respectively. These experimental results demonstrate that the performance of combining traditional word embeddings and LSTM-CRF to implement solutions for the PharmaCoNER challenge is not satisfactory. In addition to the first two methods, the others are all language model-based methods. Sun et al. [21] employed Multilingual BERT and obtain an F1-score of 89.24% during the PharmaCoNER challenge. Stoeckel et al. [19] combined a BiLSTM-CRF sequence tagger with pooled contextualized embeddings, word embeddings and sub-word embeddings using the framework FLAIR. Their method obtains an F1-score of 90.52%. Xiong et al. [18] combined Multilingual BERT, character-level representation, POS representation and word-shape representation to achieve results on the PharmaCoNER challenge. Their method obtains an F1-score of 91.05%. It can be seen that language models are of great value to the PharmaCoNER challenge. Whether it is through the use of contextualized character embeddings (e.g., Stoeckel’s work) or context word representations (e.g., Sun’s work and Xiong’s work), language models can greatly increase the ability to recognize biomedical entities in Spanish texts. Furthermore, note that all these works during the challenge were submitted blindly (i.e., the test set unknown). In this work, we employed BioBERTv1.1(+P,Cased) to generate biomedical contextualized representations to implement solutions for the PharmaCoNER task. Our method achieves the best F1-score of 92.01% from five runs, which is currently the best performance on the PharmaCoNER dataset. These experimental results show that the domain pre-training of language models is important for the PharmaCoNER task. The SOTA performance can be obtained by BioBERT using only biomedical domain knowledge and the WordPiece tokenizer.

### Performance of different BERTs

In this section, we further explore the impact of pre-training on BERT from four aspects: domain corpus, language, vocabulary, and character case. Table 5 shows the performance comparison of various BERT models. The BERT model can be regarded as a baseline model. It can be seen that the BERT model pre-trained using the biomedical domain corpus (e.g., SciBERT and BioBERT) or native language (e.g., MultiBERT and Spanish BERT) achieves higher performance than the BERT model pre-trained using the English general corpus. This experimental result shows that using the biomedical domain corpus or native language (i.e., Spanish) to pre-train BERT can improve model performance on the PharmaCoNER task. Compared with MultiBERT and Spanish BERT, the best version of SciBERT and BioBERT can obtain higher performance. This shows that domain knowledge is more helpful to improve model performance compared with the native language. Furthermore, we also observe an interesting experimental result, i.e., the performance of NCBI BERT is even lower than the original BERT. It may be caused by the large difference between the corpora of MIMIC-III and PharmaCoNER. This experimental result indicates that only the domain knowledge related to the PharmaCoNER dataset can promote the improvement of model performance. Next, we observe that all BERT models obtain competitive performance, demonstrating that BERT can take advantage of WordPiece to alleviate the OOV limitation. However, the max F1-scores of SciBERT(Scivoc,Cased) (91.05%) and SciBERT(Scivoc,UnCased) (91.15%) are higher than those of SciBERT(Bertvoc,Cased) (90.58%) and SciBERT(Bertvoc,Uncased) (90.59%). This experimental result indicates that although BERT can use WordPiece to alleviate the OOV limitation, using the vocabulary designed for the domain corpus can further improve model performance. Finally, we compare the effect of the character case on BERT models. As shown in Table 5, among these models, BERT, MultiBERT, SciBERT, and Spanish BERT have Cased and Uncased models, while NCBI BERT and BioBERT only have Uncased or Cased models. From the average F1-score, the performance of BERT (Uncased) and MultiBERT (Uncased) is better than that of BERT (Cased) and MultiBERT (Cased). However, the performance on the Cased and Uncased models is not much different for Spanish BERT and SciBERT. Therefore, as far as existing BERT models are concerned, it can only be concluded that the character case has a certain impact on model performance. The specific impact trend needs more experiments to reveal.

**Table 5:** Performance comparison of various BERTs

Method	Mean ± SD			Max		
	P (%)	R (%)	F1 (%)	P (%)	R (%)	F1 (%)
BERT(Cased)	89.31 ± 0.26	88.00 ± 0.16	88.65 ± 0.12	89.51	88.06	88.78$^*$
BERT(Uncased)	89.60 ± 0.81	88.13 ± 0.40	88.86 ± 0.57	90.32	88.65	89.48$^*$
NCBI BERT(P+M,Uncased)	89.29 ± 0.67	87.11 ± 0.60	88.18 ± 0.35	89.58	87.30	88.42$^*$
NCBI BERT(P,Uncased)	90.20 ± 0.38	88.88 ± 0.52	89.53 ± 0.37	90.76	89.58	90.16$^*$
Spanish BERT(Uncased)	89.69 ± 0.74	90.56 ± 0.58	90.12 ± 0.37	90.47	90.72	90.59$^*$
Spanish BERT(Cased)	90.42 ± 0.77	90.51 ± 0.69	90.47 ± 0.69	91.76	91.31	91.54
MultiBERT(Cased)	89.53 ± 0.27	89.99 ± 0.43	89.76 ± 0.19	89.75	90.34	90.04$^*$
MultiBERT(Uncased)	90.74 ± 0.35	90.39 ± 0.37	90.56 ± 0.25	91.02	90.77	90.89
SciBERT(Bertvoc,Cased)	90.36 ± 0.75	89.55 ± 0.30	89.96 ± 0.40	91.66	89.52	90.58$^*$
SciBERT(Bertvoc,Uncased)	91.07 ± 0.71	89.00 ± 0.45	90.02 ± 0.55	91.85	89.36	90.59$^*$
SciBERT(Scivoc,Uncased)	90.75 ± 0.86	90.27 ± 0.32	90.51 ± 0.40	92.03	90.28	91.15
SciBERT(Scivoc,Cased)	91.25 ± 0.69	90.30 ± 0.58	90.77 ± 0.40	92.40	89.74	91.05
BioBERTv1.0(+PMC,Cased)	90.54 ± 0.71	89.59 ± 0.31	90.06 ± 0.45	91.09	89.90	90.49$^*$
BioBERTv1.0(+P,Cased)	90.44 ± 0.34	89.98 ± 0.64	90.21 ± 0.36	90.75	90.55	90.65$^*$
BioBERTv1.0(+P+PMC,Cased)	91.08 ± 0.86	89.76 ± 0.52	90.41 ± 0.42	91.13	90.34	90.73
BioBERTv1.1(+P,Cased)	**91.40 ± 0.81**	**90.90 ± 0.47**	**91.15 ± 0.60**	**92.44**	**91.59**	**92.01**

‘P’ and ‘M’ denote PubMed and MIMIC-III, respectively. The table is sorted according to the average F1-score, and the highest values are shown in bold

*Significant difference between the means of two models according to the T-TEST statistical test. Specifically, it indicates the model has a significant difference compared with BioBERTv1.1(+P,Cased), with more than 95% confidence interval ($p<$ 0.05)

### Discussion

#### Performance of each type for PharmaCoNER

Table 6 lists the highest precision, recall and F1-score of BioBERTv1.1(+P,Cased) on the PharmaCoNER challenge. Among the three types of entities evaluated for PharmaCoNER, BioBERTv1.1(+P,Cased) performed worst on NO_NORMALIZABLES (16.67% in F1-score). As shown in Table 1, there are only 50 NO_NORMALIZABLES entities in the PharmaCoNER dataset. Because the quantity is insufficient, it is difficult for BioBERTv1.1(+P,Cased) to effectively learn latent features of this type of mention. In contrast, BioBERTv1.1(+P,Cased) performed well on the recognition for NORMALIZABLES and PROTEINAS entities, achieving F1-scores of 94.83% and 89.87%, respectively. The reason may be that these two types of entities are in sufficient quantity and their structure has been standardized.

**Table 6:** Performance of each type for PharmaCoNER

Method	P (%)	R (%)	F1 (%)
NORMALIZABLES	95.33	94.35	94.83
NO_NORMALIZABLES	14.29	20.00	16.67
PROTEINAS	90.45	89.29	89.87
Overall	92.44	91.59	92.01

#### Softmax versus CRF

Because CRF can optimize the path of sequence labeling problems, most previous neural models (e.g., LSTM-CRF) used CRF to learn label constraints. In this study, we compared the performance of BERT-softmax and BERT-CRF. As illustrated in Table 7, the performance of BERT-softmax is superior to that of BERT-CRF. The reason may be that the token representation already contains context information, and using these representations can obtain promising performance.

**Table 7:** Performance comparison of BERT-CRF and BERT-Softmax

Method	Mean ± SD			Max		
	P (%)	R (%)	F1 (%)	P (%)	R (%)	F1 (%)
BERT-CRF	90.42 ± 1.16	89.59 ± 0.36	90.00 ± 0.68	91.69	89.90	90.79
BERT-Softmax	**91.40 ± 0.81**	**90.90 ± 0.47**	**91.15 ± 0.60**	**92.44**	**91.59**	**92.01**

‘BERT’ refers to BioBERTv1.1(+P,Cased)

The highest values are shown in bold

#### Error analysis

We further performed error analysis to explore the entities constituting false negatives (FNs) and false positives (FPs). The best run of BioBERTv1.1(+P,Cased) (with the F1-score of 92.01%) produced a total of 155 FNs and 138 FPs. We concluded four representative types of errors by analyzing these FNs and FPs. Table 8 lists these types of errors. The first example represents a type of FNs, which is caused by incorrectly recognizing the ground-truth ‘PROTEINAS’ type as the ‘O’ type. This type of error accounts for 49% (i.e., 76/155) of all FNs. Similarly, the second example represents a type of FPs, which is caused by incorrectly recognizing the ground-truth ‘O’ type as the ‘PROTEINAS’ type. This type of error accounts for 42% (i.e., 58/138) of all FPs. Furthermore, boundary recognition errors are a typical type of error. As for the third example, the BioBERTv1.1(+P,Cased) model incorrectly recognizes some modifying words (i.e., ‘de bajo pesomolecular’) as the chemical entity (i.e., ‘heparina’). The fourth example also represents a type of error, i.e., the chemical and protein cross-recognition errors. In the gold standard, ‘urokinasa’ is annotated as a protein entity, but the BioBERTv1.1(+P,Cased) model incorrectly recognizes it as a chemical type (i.e., the ‘NORMALIZABLES’ type).

**Table 8:** Examples of errors in recognizing biomedical entities by BioBERTv1.1(+P,Cased)

	Error examples	Number of errors in this type
Gold:	Se solicita serología de **[Anticuerpos Echinococcus]**$_{(\textit{PROTEINAS})}$/Hemag que es POSITIVO a cifras superiores 1/2,621,440	76
Pred:	Se solicita serología de ***[Anticuerpos Echinococcus]***$_{(O)}$/Hemag que es POSITIVO a cifras superiores 1/2,621,440	
Gold:	A esto se añadía alteración de **[enzimas hepáticas]**$_{(O)}$	58
Pred:	A esto se añadía alteración de ***[enzimas hepáticas]***$_{(\textit{PROTEINAS})}$	
Gold:	... a dosis plenas (1 mg/kg/día) y **[heparina]**$_{(\textit{NORMALIZABLES})}$ de bajo peso molecular, con normalización progresiva de las deposiciones .	39
Pred:	... a dosis plenas (1 mg/kg/día) y ***[heparina de bajo peso molecular]***$_{(\textit{NORMALIZABLES})}$, con normalización progresiva de las deposiciones	
Gold:	La ecografía mostró derrame pleural loculado, administrándose en consecuencia 200,000 UI de **[urokinasa]**$_{(\textit{PROTEINAS})}$ durante dos días consecutivos por el tubo de toracocentesis	9
Pred:	La ecografía mostró derrame pleural loculado, administrándose en consecuencia 200,000 UI de ***[urokinasa]***$_{(\textit{NORMALIZABLES})}$ durante dos días consecutivos por el tubo de toracocentesis	

‘Gold’ denotes the gold standard, and ‘Pred’ denotes the prediction results. Bold represents the gold standard entities and bolditalic denotes the predicted entities. If not specified, it defaults to the ‘O’ type, which means it is not a chemical/protein entity

## Conclusion

In this article, we have compared and explored various representative BERTs on the PharmaCoNER dataset in detail. Our method achieves SOTA performance, with an F1-score of 92.01%. The experimental results show that the introduction of language models such as BERT can effectively improve model performance on the PharmaCoNER task. For the BERT model, the performance of the model pre-trained using the biomedical domain corpus is superior to the model pre-trained using the native language. Although BERT can use WordPiece to alleviate the OOV limitation, the use of a vocabulary designed for specific domain corpora can further improve model performance. Furthermore, the character case also has a certain effect on model performance. In future work, we would like to explore the performance of BERT pre-trained using the Spanish PubMed corpus on the PharmaCoNER dataset.

## Data Availability

The PharmaCoNER corpus can be downloaded at: https://temu.bsc.es/pharmaconer/. The transformer framework are available at: https://github.com/huggingface/transformers. The PyTorch library are available at: https://pytorch.org. Our data and codes are available at https://github.com/CongSun-dlut/PharmaCoNER.

## Abbreviations

NER: named entity recognition
NLP: natural language processing
SOTA: state-of-the-art
OOV: out-of-vocabulary
BioNER;: biomedical named entity recognition
POS: part-of-speech
LSTM: long short-term memory
CNN: covolution neural network
CRF: conditional random field
BiLSTM: bidirectional long short-term memory
SPACCC: Spanish Clinical Case Corpus.
